# Alignment efficiency and three-dimensional assessment of root resorption after alignment with conventional and copper-nickel-titanium archwires: A randomized controlled trial

**DOI:** 10.1590/2177-6709.28.6.e2323177.oar

**Published:** 2024-01-08

**Authors:** Navleen Kaur BHATIA, Vinay Kumar CHUGH, Sam Prasanth SHANKAR, Ritvik VINAY A.P, Surjit SINGH, Priyawati MOUNGKHOM, Rinkle SARDANA

**Affiliations:** 1 All India Institute of Medical Sciences, Department of Dentistry, Section of Orthodontics (Jodhpur, Rajasthan, India).; 2 All India Institute of Medical Sciences, Department of Pharmacology (Jodhpur, Rajasthan, India).

**Keywords:** Alignment, Root resorption, Fixed orthodontic appliances, Orthodontic wires, CBCT

## Abstract

**Objective:**

To compare alignment efficiency and root resorption between nickel-titanium (NiTi) and copper-nickel-titanium (CuNiTi) archwires after complete alignment in mandibular anterior region.

**Methods:**

In this two-arm parallel single-blind randomized controlled trial, forty-four patients with Class I malocclusion with mandibular anterior crowding were recruited form orthodontic clinic of All India Institute of Medical Sciences (Jodhpur, India). Patients were randomly allocated into NiTi and CuNiTi groups, with a 1:1 allocation. Alignment was performed using 0.014-in, 0.016-in, 0.018-in, 0.019x0.025-in archwire sequence in the respective groups, which terminated in 0.019 x 0.025-in stainless-steel working archwire. The primary outcome was alignment efficiency, measured on study models from baseline (T0) to the first, second, third, fourth and fifth-month (T5). Secondary outcome was root resorption, measured from CBCT scans taken at T0 and T5. Mixed-factorial ANOVA was used to compare Little’s Irregularity Index (LII). For assessing the proportion of patients with complete alignment at the end of each month, Kaplan-Meier survival curve was built and time to treatment completion was compared between groups using log rank test. Paired *t*-test was used to assess external apical root resorption (EARR) within groups, whereas independent *t*-test was used to evaluate LII and EARR between the groups.

**Results:**

Twenty-two patients were recruited in each group. One patient was lost to follow-up in the CuNiTi group. No statistically significant differences were observed in alignment efficiency between the groups (*p*>0.05). Intergroup comparison revealed that the changes in root measurement in three-dimensions were not statistically significant (*p*>0.05), except for mandibular right central incisor, which showed increased resorption at root apex in NiTi group (*p*<0.01).

**Conclusion:**

The two alignment archwires showed similar rate of alignment at all time points. Root resorption measurement did not differ between the NiTi and CuNiTi groups, except for the mandibular right central incisor, which showed more resorption in NiTi group.

## RESUMO

**Objetivo:**

Comparar os fios de níquel-titânio (NiTi) e de cobre-níquel-titânio (CuNiTi) quanto à eficiência do alinhamento e quantidade de reabsorção radicular, após alinhamento completo dos dentes da região anterior inferior.

**Métodos:**

Neste estudo clínico randomizado, cego, paralelo, de dois braços, quarenta e quatro pacientes com má oclusão Classe I e apinhamento anterior inferior foram recrutados na clínica ortodôntica do All India Institute of Medical Sciences (Jodhpur, India). Os pacientes foram alocados aleatoriamente nos grupos NiTi e CuNiTi, na proporção de 1:1. O alinhamento foi realizado usando a sequência de fios 0,014”, 0,016”, 0,018” e 0,019” x 0,025” nos respectivos grupos, finalizando com o arco de trabalho 0,019” x 0,025” de aço inoxidável. O desfecho primário foi a eficiência do alinhamento, medida nos modelos de estudo nos tempos inicial (T0) e após um, dois, três, quatro e cinco meses (T5). O desfecho secundário foi a reabsorção radicular, medida a partir de tomografias computadorizadas realizadas em T0 e T5. ANOVA fatorial mista foi utilizada para comparar o Índice de Irregularidade de Little (IIL). Para avaliar a proporção de pacientes com alinhamento completo ao fim de cada mês, foi construída uma curva de sobrevida pelo método de Kaplan-Meier, e o tempo até o fim do tratamento foi comparado entre os grupos por meio do teste *log-rank*. Um teste *t* pareado foi utilizado para avaliar a reabsorção radicular apical externa (RRAE) dentro dos grupos, enquanto um teste *t* independente foi utilizado para avaliar o IIL e a RRAE entre os grupos.

**Resultados:**

Vinte e dois pacientes foram recrutados em cada grupo. Um paciente perdeu o acompanhamento no grupo CuNiTi. Não foram observadas diferenças estatisticamente significativas entre os grupos quanto à eficiência do alinhamento (*p*>0,05). A comparação intergrupos revelou que as alterações na RRAE medida em três dimensões não foram estatisticamente significativas (*p*>0,05), exceto para o incisivo central inferior direito, que apresentou aumento da RRAE no grupo NiTi (*p*<0,01).

**Conclusão:**

Os dois tipos de fios de alinhamento apresentaram taxa de alinhamento semelhante em todos os momentos. A medida da reabsorção radicular não diferiu entre o grupo NiTi e CuNiTi, exceto para o incisivo central inferior direito, que apresentou maior reabsorção no grupo NiTi.

## INTRODUCTION

Clinically effective treatment aims at balancing light, continuous forces and the restriction of potential damage to the tooth and periodontal structures.^1^ In the first stage of orthodontic treatment, alignment archwires should have the property of delivering light continuous forces for a longer period of time. Nickel-titanium (NiTi) and copper-nickel-titanium (CuNiTi) alloys are common archwire choices to achieve ideal alignment of teeth.^2^ Quintão et al.^3^ stated that the smaller force deflection of superelastic NiTi archwires, when compared with stainless steel ones, makes the former more favorable for correction of crowding. However, despite the availability of various archwires, a careful selection should be made, due to the differences in expression of their properties *in vivo*.^1^

A light continuous force is usually desired throughout the fixed orthodontic treatment. Dalaie et al.^4^ have shown that there is no adverse effect of leveling and alignment on root length of immature teeth. However, heavy orthodontic forces may lead to potential side-effects like pain and root resorption. According to Tripuwabhrut et al.,^5^ orthodontic forces represent a physical agent that is itself capable of inducing inflammatory reaction in the periodontium. Orthodontically induced external apical root resorption (EARR) is one of the most commonly seen iatrogenic damage of fixed orthodontic treatment, which is a result of inflammation caused by excessive forces. Various diagnostic modalities are available to evaluate root resorption.^6^ ^-^ ^8^ Cone beam computed tomography (CBCT) appears to be a better and reliable tool to assess root resorption with marked accuracy.^9^ It has been used to assess EARR after rapid maxillary expansion,^10^ ^,^ ^11^ intrusion movements,^12^ ^,^ ^13^ initial alignment and leveling,^7^ ^,^ ^14^ ^,^ ^15^ and after complete orthodontic treatment.^16^ Patients with detectable root resorption during the first six-months of active treatment have been reported to have greater resorption in the following six-month period.^16^ ^,^ ^17^

Previous studies comparing different NiTi, CuNiTi or multi-stranded stainless-steel (SS) archwires did not find any significant differences in terms of alignment efficiency and EARR.^18^ ^-^ ^24^ However, there is insufficient data to make clear recommendations regarding the superiority of any available archwire, in relation to their effectiveness and efficacy, due to the small number of robust *in-vivo* studies.^24^ A Cochrane review stated that more extensive studies are needed to formulate specific guidelines.^25^ The studies evaluating root resorption after the initial phase of alignment using CBCT have assessed changes in root length mainly in sagittal section. Comprehensive three-dimensional assessment of EARR in all sections after alignment and leveling has been rarely reported. The present study is probably the first to compare three-dimensional (sagittal, coronal and axial section) changes in root measurement using CBCT between NiTi and CuNiTi groups. 

The primary outcome of this study was to compare the alignment efficiency. Three-dimensional assessment of root resorption was the secondary outcome. The null hypothesis was that there would be no difference in alignment efficiency and EARR after complete alignment between NiTi and CuNiTi archwires. 

## MATERIAL AND METHODS

### TRIAL DESIGN

The study was an open-label, parallel-group and randomized clinical trial, with a 1:1 allocation ratio. No changes were made in methodology after trial commencement.

### PARTICIPANTS, ELIGIBILITY CRITERIA, AND SETTINGS

**Figure 1:**
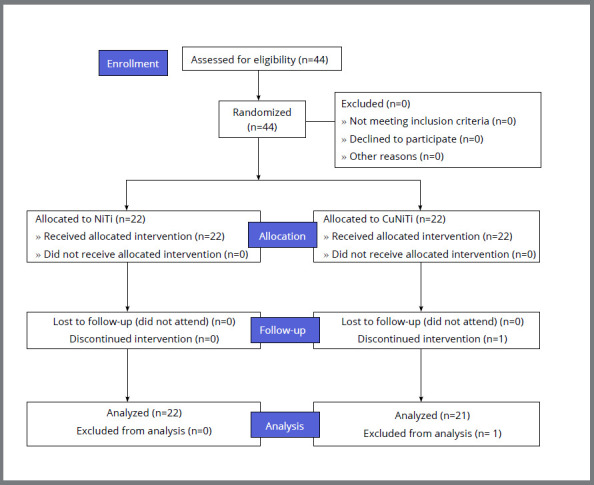
CONSORT flowchart of participants through each stage of the trial.

Ethical approval was obtained (AIIMS/IEC/2018/610) from the Institutional Ethics Committee, All India Institute of Medical Sciences (AIIMS, Jodhpur, India). This study was also registered at Clinical Trials Registry, India (CTRI/2018/10/016038). The CONSORT statement reporting guidelines were followed (Fig 1). The recruitment of orthodontic patients was done at the post-graduate orthodontic clinic, Department of Dentistry, AIIMS (Jodhpur, India), between July 2018 and March 2020. Forty-four patients who fulfilled the inclusion criteria were invited to participate in the study. Patients having Angle’s Class I malocclusion with moderate (4-6 mm) to severe (>6 mm) mandibular anterior crowding, based on the contact point displacement, were recruited for the study.^26^ The inclusion and exclusion criteria of the participants are shown in Table 1. 

**Table 1:** Selection criteria.

Inclusion criteria
Age 12 years and older at the beginning of fixed orthodontic treatment
Moderate (4-6 mm) to severe (>6 mm) mandibular anterior crowding requiring extraction of mandibular first premolars
Mandibular permanent dentition, excluding third molars
Absence of root resorption in mandibular anterior teeth
Absence of any impacted teeth in the mandibular anterior region
Exclusion criteria
Any history of previous orthodontic treatment
Treatment plan involving single mandibular incisor extraction
Completely blocked-out mandibular incisor, in which engagement of the first archwire was not possible
Poor gingival and periodontal health
Patients with any systemic, bone or metabolic disorders
Under medication that alter tooth movements

### RANDOMIZATION, ALLOCATION CONCEALMENT AND BLINDING

Patients were randomly allocated into two groups using computer-generated variable block randomization scheme, in a specific software (Random Allocation Software 2.0, Microsoft Corporation, WA, USA). The allocation concealment was achieved using an opaque sealed envelope (sequentially numbered as per randomization scheme). Operator was handed over these envelopes by a person not involved in the trial. The outcome assessor, participants and the statistician were blinded; however, the operator could not be blinded to the allocation groups. The information of the participants was anonymized using non-identifiable codes and removing identifying information.

### INTERVENTIONS

All participants were bonded with fixed appliances in both, maxillary and mandibular arches (Gemini brackets, 0.022-in MBT preadjusted Edgewise prescription; 3M Unitek, Monrovia, Calif). After performing atraumatic extractions of the mandibular first premolars, leveling and alignment was initiated using NiTi archwire (G4™ Nickel-titanium, G&H, Franklin, Indiana) in the NiTi group, and CuNiTi archwire (M5™ Thermal Copper-Nickel-Titanium 35ºC, G&H, Franklin, Indiana) in the CuNiTi group. The crowding measured from the initial data was recorded as T0, at the baseline. In both groups, archwires were ligated in the sequence as shown in Table 2. At the end of every month, archwires were changed sequentially for five-months, until the placement of 0.019 × 0.025-in SS working archwire in both groups, to produce a common end-point (T5). Lacebacks to canines were used to obtain space after extraction for alleviation of crowding. N.K.B. performed all the clinical interventions relating to the study. S.P.S. measured the outcome variables. 

**Table 2:** Time period and archwire sequence used in the study.

Time period	Archwire sequence
T0 (At the baseline)	Placement of fixed appliances and 0.014-in archwire
T1 (At the end of first month)	Removal of 0.014-in archwire and placement of 0.016-in archwire
T2 (At the end of second month)	Removal of 0.016-in archwire and placement of 0.018-in archwire
T3 (At the end of third month)	Removal of 0.018-in archwire and placement of 0.019 × 0.025-in archwire
T4 (At the end of fourth month)	Removal of 0.019×0.025-in archwire and placement of working 0.019×0.025-in stainless-steel archwire
T5 (At the end of fifth month)	Completion of the alignment phase

### OUTCOMES

To assess alignment efficiency, mandibular impressions were taken at the beginning of fixed orthodontic treatment and thereafter every month, up to five-months of treatment. CBCT scan of mandibular anterior region were obtained at pre-treatment and post-alignment stage. The scan was obtained using a using a CBCT unit (KODAK 9600 3D^®^; Carestream Health, Inc., Marne-la-Vallée, France), with reduced field of view (5x5cm)^27^ and voxel size of 0.15mm, as suggested by Samandara et al.^9^, with standard recommended settings (voltage = 120kV; current = 8mA; scan time = 10s). The data was then imported into Dolphin imaging software (version 11.95, Dolphin Imaging & Management Solutions, Chatsworth, Calif).

» Primary outcomes - Alignment efficiency was analyzed assessing the reduction in the Little’s irregularity index (LII)^28^, to measure crowding of mandibular anterior region, on study models obtained at T0 to T5. Displacement of contact point as described in the index was measured with the help of a digital caliper with a sharpened fine edge to the nearest 0.01 mm (Standard Digital Caliper Series: EC16) on the study models.^19^ ^,^ ^29^ ^,^ ^30^ Alignment was considered complete when LII was near zero and 0.019×0.025-in stainless-steel working archwire was passive in the bracket slots (T5).

**Figure 2:**
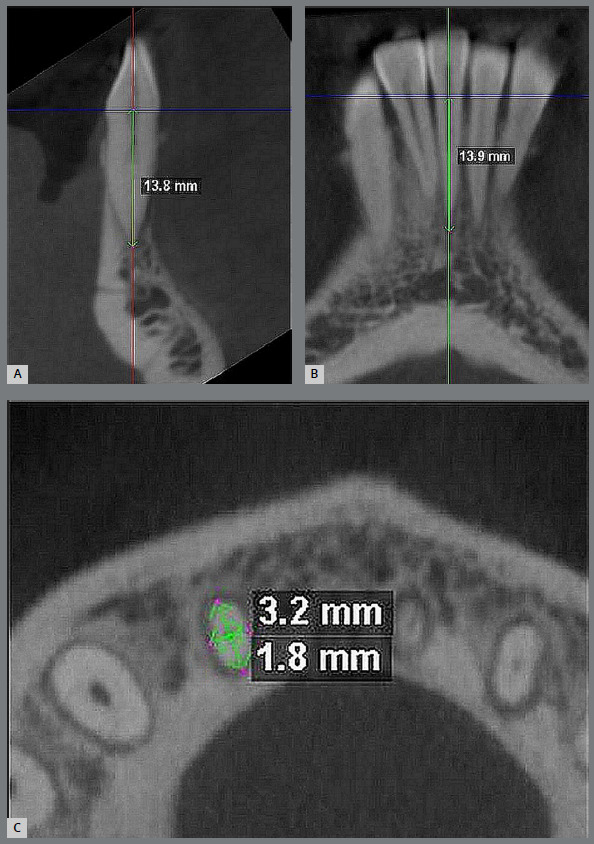
A) Sagittal section; B) coronal section; C) axial section showing labio-lingual thickness and mesio-distal width.

» Secondary outcomes - Changes in the root length and apical root dimensions were assessed for the four mandibular incisors at T0 and T5 in CBCT scans of mandibular anterior region, as per measurements described in Table 3 and Figure 2.

**Table 3:** Measurement used for evaluation of root resorption.

Root length (mm)
The scan was adjusted along the long axis for the tooth to be measured, such that the horizontal reference line of software passes through cemento-enamel junction (CEJ). The length of the root was measured along the long axis of the tooth, passing from the most apical point of the root to the reference line perpendicularly. The root length measurements were made in sagittal and coronal sections for the four mandibular incisors (Fig 2A-B).
Apical root dimension (mm)
The root length was measured from CEJ to the apex, and marked 2mm short of apex using ‘landmark’ tool in the coronal section in pre-treatment scans (T0). Axial slice was viewed at this level and labio-lingual and mesio-distal dimensions were measured to determine apical root diameter. Post-alignment scans (T5) were evaluated at the same root level from CEJ in the coronal section as T0, for the apical root measurements (Fig 2C).

### SAMPLE SIZE

Sample size was calculated based on the previous study by Serafim et al.^30^ Assuming a proportion of 73% and 100% patients achieving complete alignment in the two groups, and population risk difference of 0.2, a sample size of 19 patients per group was calculated. Clinical significance level of 0.05 was established. Considering a dropout rate of about 10%, we recruited 22 patients per treatment group. Sample size was calculated using nMaster 2.0 software.

### INTERIM ANALYSES AND STOPPING GUIDELINES

Not applicable.

### STATISTICAL ANALYSIS

Data were analyzed using IBM SPSS 23.0 (Armonk, NY: IBM Corp.). One calibrated blinded examiner (S.P.S.) evaluated the study models and CBCT scans. Dahlberg error^26^ and intra-examiner reproducibility (repeated after two weeks) were assessed using intraclass correlation coefficients (ICC) for LII and all measurements for EARR. 

Normal distribution was assessed using Shapiro-Wilk tests. Descriptive statistics of means and standard deviations (SD) were calculated. Chi-square test/Fisher’s Exact test was applied to compare baseline categorical data. Mixed factorial ANOVA was used to compare differences in the alignment between the groups with time interaction. Kaplan-Meier survival curve was built to illustrate proportion of patients who completed alignment at the end of each month. Log-rank test was used to compare time to treatment completion between groups. Paired *t*-test was used to assess EARR, before and after alignment within groups, and an independent *t*-test was used to evaluate the difference in LII and EARR between the groups. The significance level was set as *p*<0.05, with 95% confidence intervals (95% CI). 

## RESULTS

### PARTICIPANT FLOW

Forty-four patients were randomly allocated in a 1:1 ratio into NiTi and CuNiTi groups. One patient was lost to follow-up after randomization in the CuNiTi group, and was excluded of the analysis. A CONSORT diagram showing the flow of participants during the trial is presented in the Figure 1. Dahlberg error for LII and EARR indicated no significant measurement error (LII < 0.4 mm; EARR = 0.48-0.55mm). Intra-examiner reproducibility of the main examiner was excellent for LII and good to excellent for all measurements regarding EARR (Supplemental Table).

**Supplemental Table:** Intraclass correlation coefficients for intra-examiner reproducibility and inter-examiner reliability.

Parameters	ICC (95% CI)			
	Intra-examiner reproducibility	P-value*	Inter-examiner reliability	P-value^#^
Study models				
Little’s Irregularity Index	0.973 (0.917 - 0.992)	< 0.001**	0.978 (0.923-0.993)	< 0.001**
CBCT scans				
Root length-sagittal section	0.894 (0.681 - 0.965)	< 0.001**	0.959 (0.858 - 0.987)	< 0.001**
Root length-coronal section	0.908 (0.719 - 0.970)	< 0.001**	0.975 (0.922 - 0.992)	< 0.001**
Labiolingual thickness-axial section	0.898 (0.643 - 0.968)	< 0.001**	0.936 (0.797 - 0.979)	< 0.001**
Mesiodistal width-axial section	0.956 (0.737- 0.988)	< 0.001**	0.883 (0.388 - 0.968)	< 0.001**

* P-value representing intra-examiner reproducibility. ^#^P-value representing and inter-examiner reliability ** P-value < 0.05 is considered as significant; Intraclass correlation was analyzed using two-way mixed model, with absolute agreement.

### BASELINE DATA

Table 4 presents the baseline characteristics of the patients in each treatment group. NiTi group consisted of twenty-two patients (8 males, 14 females), whereas CuNiTi group consisted of twenty-one patients (8 males, 13 females). There was no significant difference in the baseline parameters between the groups. 

**Table 4:** Baseline characteristics of participants in each study group.

Variable	NiTi n=22	CuNiTi n=21	Significance
Initial age (years) mean (SD)	16.9 (3.63)	17.4 (4.64)	NS
Sex - subjects n (%)			
Male	8 (36.4)	8 (38.1)	NS
Female	14 (63.6)	13 (61.9)	
Crowding - subjects n (%)			
Moderate	10 (45.5)	10 (47.6)	NS
Severe	12 (54.5)	11 (52.4)	
LII (mm) mean (SD)	6.2 (2.42)	6.4 (3.32)	NS

Values are presented as mean (SD), or n (%). SD = standard deviation, NiTi = nickel-titanium, CuNiTi = copper-nickel-titanium, LII = Little’s irregularity index, NS = non-significant.

### PRIMARY OUTCOME

**Figure 3:**
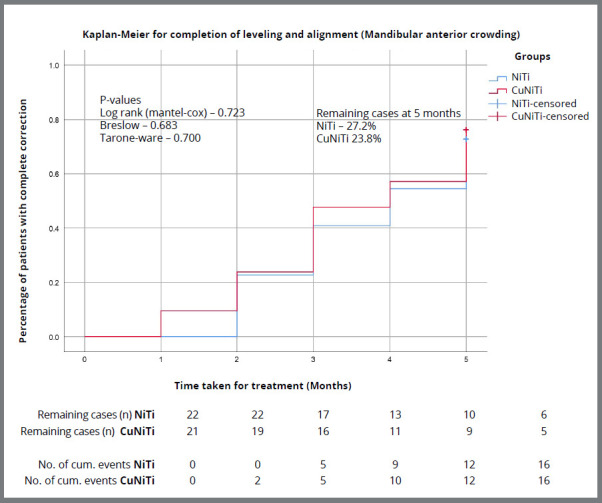
Kaplan-Meier survival curves for the two groups used in the study. The y-axis gives the proportion of patients achieving complete alignment at different time points ( months on x axis ). There is no separation during the evaluation period, indicating no significant difference between the groups.

There was a significant improvement in LII after every month in NiTi and CuNiTi groups; however, the difference in LII with time was not significant (*p*>0.05) between the two groups (Tables 5 and 6). The log-rank test revealed no statistically significant difference between the two types of alignment archwires (*p*>0.05) in terms of survival function (Fig 3). No statistically significant difference (*p*>0.05) was present in both the groups, in terms of number of patients in which alignment was incomplete at the end of five months (Table 7).

**Table 5:** Mixed factorial ANOVA test for the difference between groups, according to the change in LII with time factor.

Source	F	P value
Time	140.809	<0 .001*
Time * Type of wire	0.639	0.502
Type of wire	0.146	0.704

LII = Little’s Irregularity Index; *P < 0.05 = statistically significant.

**Table 6:** Comparison of alignment efficiency using LII (mm) between NiTi and CuNiTi groups at different time intervals.

Measurement	NiTi (n=22)		CuNiTi (n=21)		***p*-value**	95% CI	
	Mean	SD	Mean	SD		Lower bound	Upper bound
LII (mm)							
At T0	6.2	2.42	6.3	3.28	0.833	-1.94	1.57
T0-T1	3.8	1.81	3.3	2.56	0.432	-0.83	1.91
T1-T2	2	1.82	1.6	1.53	0.411	-0.61	1.46
T2-T3	1	1.28	0.8	0.93	0.576	-0.50	0.88
T3-T4	0.3	0.46	0.5	0.67	0.419	-0.49	0.21
T4-T5	0.2	0.32	0.1	0.22	0.538	-0.12	0.22

Values are presented as mean (SD). SD = standard deviation, NiTi = nickel-titanium, CuNiTi = copper-nickel-titanium, LII = Little’s irregularity index, CI = confidence interval; P-value for comparison of group means by independent t-test.

**Table 7:** Comparison of proportion of cases showing completion of alignment at T5, in each group.

Variable	NiTi n=22	CuNiTi n=21	P value*	Odds Ratio (OR) (95% CI)
Alignment completed/incomplete n/n (%) after 5 months	16/6 (72.8/27.2)	16/5 (77.3/22.7)	1.000	1.2 (0.304-4.743)

NiTi = nickel-titanium; CuNiTi = copper-nickel-titanium CI = Confidence Interval *p-value for comparison of percentage data by Fischer’s Exact test CI-Mantel-Haenszel Common odds ratio.

### SECONDARY OUTCOME

The intragroup comparison showed significant differences (*p*<0.05) in root length and apical root dimension (labio-lingual and mesio-distal measurements) in all four mandibular incisors in both groups (Table 8). However, the differences between the two groups were not statistically significant (*p*>0.05) for all measurements, except for the mandibular right central incisor, which showed significantly higher resorption in NiTi group for apical root dimension, both in mesio-distal and labio-lingual dimension (Table 9). 

**Table 8:** Comparison of linear changes in root length from T0-T5 (mm) in NiTi and CuNiTi groups.

Measurement	NiTi (n=22)				CuNiTi (n=21)			
	T0 Mean (SD)	T5 Mean (SD)	Mean diff	P value	T0 Mean (SD)	T5 Mean (SD)	Mean diff	P value
Root length - SS								
LR1	12.5 (1.21)	12.0 (1.34)	0.6	<0.001*	12.5 (0.83)	12.0 (0.93)	0.5	<0.001*
LL1	12.5 (1.24)	12.1 (1.34)	1.0	<0.001*	12.5 (0.97)	12.0 (1.11)	0.5	<0.001*
LR2	13.7 (1.33)	12.6 (1.32)	1.0	<0.001*	13.7 (1.09)	12.8 (1.24)	0.8	<0.001*
LL2	13.8 (1.27)	12.7 (1.28)	0.4	<0.001*	13.7 (1.15)	12.9 (1.18)	0.8	<0.001*
Root length - CS								
LR1	12.5 (1.51)	11.2 (1.33)	1.2	<0.001*	12.6 (0.89)	11.9 (1.09)	0.7	<0.001*
LL1	12.5 (1.48)	11.3 (1.52)	1.2	<0.001*	12.6 (0.95)	11.7 (1.18)	0.8	<0.001*
LR2	13.5 (1.44)	12.1 (1.37)	1.4	<0.001*	13.7 (1.13)	12.8 (1.25)	0.9	<0.001*
LL2	13.4 (1.44)	12.1 (1.48)	1.3	<0.001*	13.7 (1.14)	12.7 (1.41)	0.9	<0.001*
Root apex (LL) - AS								
LR1	3.2 (0.80)	2.1 (1.64)	1.2	<0.001*	3.4 (0.43)	3.0 (0.89)	0.3	0.035 ***
LL1	3.3 (0.29)	2.2 (1.64)	1.0	0.001*	3.6 (0.56)	2.9 (1.15)	0.6	0.004 **
LR2	3.1 (0.77)	1.8 (1.56)	1.2	<0.001*	3.6 (0.90)	2.7 (1.49)	0.8	0.003 **
LL2	3.3 (0.90)	2.1 (1.63)	1.2	<0.001*	3.5 (0.78)	2.7 (1.06)	0.8	0.001*
Root apex (MD) - AS								
LR1	1.6 (0.19)	1.0 (0.66)	0.6	<0.001*	1.5 (0.22)	1.3 (0.38)	0.2	0.026 ***
LL1	1.5 (0.23)	1.1 (0.65)	0.4	0.003 **	1.6 (0.20)	1.4 (0.45)	0.2	0.004 **
LR2	1.5 (0.22)	0.9 (0.73)	0.6	<0.001*	1.6 (0.23)	1.1 (0.53)	0.5	0.001*
LL2	1.6 (0.30)	1.0 (0.68)	0.6	<0.001*	1.6 (0.20)	1.3 (0.43)	0.3	0.005 **

Values are presented as mean (SD). SD = standard deviation, diff = difference; T0 = pre-alignment, T5 = post-alignment, NiTi = nickel-titanium, CuNiTi = copper-nickel-titanium, LR1 = mandibular right central incisor LL1 = mandibular left central incisor, LR2 = mandibular right lateral incisor, LL2 = mandibular left lateral incisor, SS = sagittal section, CS = coronal section, AS = axial section, LL = labio-lingual thickness, MD = mesio-distal width. *p*-value for comparison of group means by paired t-test.

* P < 0.001, ** P < 0.01, *** P < 0.05.

**Table 9:** Comparison of linear changes in root length from T0-T5 (mm) between NiTi and CuNiTi groups.

Measurement	NiTi (n=22) Mean (SD)	CuNiTi (n=21) Mean (SD)	P value	95% CI	
				Lower bound	Upper bound
Root length - SS					
LR1	0.6 (0.34)	0.5 (0.43)	0.322	-2.36	7.02
LL1	1.0 (0.70)	0.5 (0.43)	0.293	-1.59	5.13
LR2	1.0 (0.88)	0.8 (0.69)	0.693	-4.22	2.83
LL2	0.4 (0.36)	0.8 (0.69)	0.181	-7.95	1.54
Root length - CS					
LR1	1.2 (1.13)	0.7 (0.77)	0.698	-0.19	0.28
LL1	1.2 (1.25)	0.8 (0.78)	0.366	-0.35	0.13
LR2	1.4 (1.32)	0.9 (0.82)	0.267	-0.21	0.75
LL2	1.3 (1.36)	0.9 (0.76)	0.191	-0.14	0.69
Root apex (LL) - AS					
LR1	1.2 (1.28)	0.3 (0.67)	0.008 **	0.23	1.47
LL1	1.0 (1.26)	0.6 (0.91)	0.214	-0.25	1.09
LR2	1.2 (1.25)	0.8 (1.16)	0.294	-0.35	1.12
LL2	1.2 (1.27)	0.8 (0.99)	0.273	-0.31	1.07
Root apex (MD) - AS					
LR1	0.6 (0.64)	0.2 (0.33)	0.008 **	0.12	0.74
LL1	0.4 (0.60)	0.2 (0.33)	0.169	-0.09	0.50
LR2	0.6 (0.64)	0.5 (0.56)	0.440	-0.22	0.50
LL2	0.6 (0.64)	0.3 (0.42)	0.070	-0.03	0.63

Values are presented as mean (SD). SD = standard deviation, T0 = pre-alignment, T5 = post-alignment, NiTi = nickel-titanium, CuNiTi = copper-nickel-titanium, LR1 = mandibular right central incisor, LL1 = mandibular left central incisor, LR2 = mandibular right lateral incisor, LL2 = mandibular left lateral incisor, CI = confidence interval, SS = sagittal section, CS = coronal section, AS = axial section, LL = labio-lingual thickness, MD = mesio-distal width. P-value for comparison of group means by independent t-test: * P<0.001, ** P<0.01, *** P<0.05.

### HARMS

No adverse events were reported during treatment, except a slight EARR that was within the limits of usual occurrence during orthodontic treatment. 

## DISCUSSION

### ALIGNMENT EFFICIENCY

On evaluating alignment efficiency with both the archwires, LII scores approached zero after five-months with similar rate of alignment at each time-point. This shows that NiTi and CuNiTi were similar regarding their alignment efficiency to relieve moderate to severe crowding. Previous studies found no significant difference regarding alignment efficiency between CuNiTi and other NiTi archwires (conventional and superelastic).^20^ ^,^ ^21^ ^,^ ^31^ ^,^ ^32^ Riley and Bearn^33^ conducted a systematic review and found inadequate evidence for determining the most effective archwire for alignment. In terms of alignment efficiency and sequence of aligning archwires, similar findings were reported by a meta-analysis by Papageorgiou et al.^24^ A recent Cochrane review found no sufficient evidence to substantiate superiority of any archwire material for alignment and levelling.^25^ This review identified twelve studies, out of which only two studies compared conventional NiTi with CuNiTi, and none of them used rectangular archwires in the sequence - and therefore, may not contribute to greater evidence. The present study used conventional MBT bracket system, which was not used in both of these studies. The authors suggested a need of well-designed randomized control trial to evaluate the effectiveness of any archwire, due to presence of existing low quality of evidence.^25^ Therefore, all of the previous systematic reviews highlighted the requirement of a robust and well-designed randomized control trial to evaluate efficiency of various archwires. 

Previous studies by Abdelrahman et al.^21^ and Pandis et al.^20^ evaluated alignment efficiency using only a single round archwire for the entire duration of the study. In the former study, 0.014-in NiTi archwire was used and an impression was taken at every 2-week for 16-weeks, while in the latter 0.016-in NiTi archwire was evaluated monthly for six months without any further change in the archwire. We believe that evaluation of alignment at every 2-weeks may not offer any additional advantage, as a significant tooth movement is unlikely to occur in such a short period. Recall visits at three weeks or less may be insufficient for archwires to completely express their alignment efficiency. In the present study, the archwires were changed after every month, which is commonly accepted.^34^ Although the results of the present study are in agreement with previous studies, there is a lot of variation in evaluation periods (ranging between 2-weeks to 6-9 months) and the type and sequence of archwires used in the literature.^20^ ^,^ ^21^ ^,^ ^31^ ^,^ ^32^

The present study used both round and rectangular archwire sequences for complete alignment. Most studies^18^ ^,^ ^20^ ^,^ ^21^ ^,^ ^31^ ^,^ ^32^ in the past included only round wires and very few reported the use of both round and rectangular wires in evaluation of alignment efficiency.^22^ ^,^ ^30^

In the present study, the majority of the patients completed alignment within five months, with no statistically significant differences between the groups. Nearly 72.7% of subjects in NiTi group and 77.3% in CuNiTi group achieved complete alignment by the end of five months, which was in contrast with previous studies. Serafim et al.^30^ reported complete alignment in 70% of cases in NiTi group and 100% of cases in CuNiTi group at the end of five months; however, mechanical properties of archwire may vary with different brands and therefore, needs to be evaluated in future studies.

It appears from the ongoing discussion that NiTi and CuNiTi archwires are similar with respect to alignment efficiency, although clinically, CuNiTi may offer the advantage of better engagement in severely displaced teeth.

### ROOT RESORPTION

The significance of assessing root resorption at the initial phase of the treatment is that the teeth that show EARR in first 6-months of appliance placement are likely to present greater resorption by the end of treatment.^17^ Wang et al.^25^ suggested more randomized trials with sufficient duration, in which the benefits and possible disadvantages associated with different types of the archwires currently being used should be reported. 

In the past, periapical radiographs were commonly used to study root resorption; however, there have inaccuracies associated with it.^35^ ^,^ ^36^ Lund et al.^15^ found a slanting type of root resorption in many teeth, which can only be evaluated in three-dimensional radiography. Alamadi et al.^36^ in their study also found that two-dimensional radiographs, such as periapical radiographs and panoramic radiographs, underestimated root resorption, especially the slanting type of apical resorption, when compared to three-dimensional imaging methods. For detection of root resorption, three-dimensional imaging is considered superior to conventional imaging methods. The radiation exposure associated with CBCT scans may be considered a potential disadvantage for the patients; however, the newer CBCT techniques have presented reduced radiation exposure, compared to conventional machines.^37^ ^,^ ^38^ To Samandara et al.,^9^ a voxel size of less than 0.2-mm is considered accurate for evaluating root resorption; therefore, a voxel size of 0.15 mm was used for evaluating root resorption in the present study, which enabled visualization of small changes in root surface.

Sagittal and coronal slices of CBCT showed a significant EARR in all mandibular incisors post-alignment, irrespective of the archwire used, which is also supported by the results of previous studies.^16^ ^,^ ^21^ A recent randomized control trial, corroborating the present study, also did not find any significant difference in EARR in mandibular incisors while comparing superelastic NiTi and heat-activated NiTi archwires.^29^ In a study by Neves et al.,^39^ the incidence of EARR was reported to be higher in patients treated with premolar extraction, although the results were based on two-dimensional periapical radiographs and involved only maxillary incisors. 

There were no statistically significant differences in labiolingual and mesiodistal dimension in axial section between the two groups, except for the mandibular right central incisor, in which a significantly higher EARR was reported in NiTi group, both labio-lingually and mesio-distally, which was quite an unusual finding. A possible explanation could be the higher uncontrolled tipping produced in this particular tooth with the NiTi archwires. A previous study reported similar findings, in which mandibular left central incisor showed significantly more EARR while comparing superelastic NiTi with multi-stranded stainless-steel archwires.^6^ This may suggest a greater tendency for EARR in the NiTi archwires with a longer treatment duration. In the present study, we assessed mandibular incisors, which are the most commonly affected teeth after maxillary incisors.^11^ Direct comparison with previous studies was difficult, as most of the studies evaluated root resorption using either different type of appliances,^40^ different archwires and their sequences,^18^ ^,^ ^29^ assessment time,^16^ ^,^ ^18^ ^,^ ^29^ imaging software or type of imaging modality.^41^ ^,^ ^42^ Future studies can benefit from the uniformity in the method and timing of assessment of EARR. The results of the present study showed a significant root resorption observed in the alignment stage; therefore, a regular radiographic follow-up of the patients undergoing fixed orthodontic treatment is suggested.

## STRENGTHS AND LIMITATIONS

Every patient enrolled in this trial was followed-up for a period of five months, which gave adequate opportunity to evaluate alignment. Additionally, EARR was truly studied in all three-dimensions. One of the limitations of the current study was that the interventions could have been evaluated in non-extraction patients as well. At present, the available literature shows a lot of variation in three-dimensional assessment of root resorption; therefore, a standard method of assessment will facilitate meaningful comparisons associated with various appliances and treatment mechanotherapy. The results of the study cannot be generalized, as it was based on a single-center study. Since the limitations were fairly minor, without any discernible effect on the primary or secondary outcomes, the objectives of the study were achieved, with minimal impact from the above limitations. 

## CONCLUSIONS

The following conclusions were drawn from the study:

1. Alignment efficiency did not differ significantly between NiTi and CuNiTi groups.
2. There were no statistically significant differences in root resorption in all three-dimensions between the two groups, except for mandibular right central incisor, which showed increased resorption in root apical dimension in NiTi group, when compared to the CuNiTi group.
